# Identifying the Steps Required to Effectively Implement Next-Generation Sequencing in Oncology at a National Level in Europe

**DOI:** 10.3390/jpm12010072

**Published:** 2022-01-08

**Authors:** Denis Horgan, Giuseppe Curigliano, Olaf Rieß, Paul Hofman, Reinhard Büttner, Pierfranco Conte, Tanja Cufer, William M. Gallagher, Nadia Georges, Keith Kerr, Frédérique Penault-Llorca, Ken Mastris, Carla Pinto, Jan Van Meerbeeck, Elisabetta Munzone, Marlene Thomas, Sonia Ujupan, Gilad W. Vainer, Janna-Lisa Velthaus, Fabrice André

**Affiliations:** 1 European Alliance for Personalised Medicine, Avenue de l’Armee/Legerlaan 10, 1040 Brussels, Belgium; 2 European Institute of Oncology, IRCCS, Via Giuseppe Ripamonti, 435, 20141 Milan, Italy; giuseppe.curigliano@ieo.it (G.C.); elisabetta.munzone@ieo.it (E.M.); 3 Department of Oncology and Hemato-Oncology, University of Milan, Via Festa del Perdono, 7, 20122 Milan, Italy; 4 Institute of Medical Genetics and Applied Genomics, University of Tuebingen, Calwerstrasse 7, 72070 Tuebingen, Germany; olaf.riess@med.uni-tuebingen.de; 5 Laboratory of Clinical and Experimental Pathology, University of Côte d’Azur, FHU OncoAge, Biobank BB-0033-00025, Pasteur Hospital, 30 Avenue de la voie Romaine, CEDEX 01, 06001 Nice, France; hofman.p@chu-nice.fr; 6 Institute for Pathology, University Hospital Cologne, Kerpener Str. 62, 50937 Cologne, Germany; reinhard.buettner@uk-koeln.de; 7 The Veneto Institute of Oncology, IRCCS, Via Gattamelata, 64, 35128 Padua, Italy; pierfranco.conte@unipd.it; 8 Department of Surgical, Oncological and Gastroenterological Sciences, University of Padua, Via Giustiniani, 2, 35124 Padua, Italy; 9 Medical Faculty, University of Ljubljana, Vrazov trg 2, 1000 Ljubljana, Slovenia; tanja.cufer@mf.uni-lj.si; 10 School of Biomolecular and Biomedical Science, University College Dublin, Belfield, D04 V1W8 Dublin, Ireland; William.Gallagher@ucd.ie; 11 Exact Sciences, Quai du Seujet 10, 1201 Geneva, Switzerland; ngeorges@exactsciences.com; 12 School of Medicine and Dentistry, University of Aberdeen, Foresterhill, Aberdeen AB25 2ZD, UK; k.kerr@abdn.ac.uk; 13 Centre Jean Perrin, 58, Rue Montalembert, CEDEX 01, 63011 Clermont-Ferrand, France; Frederique.PENAULT-LLORCA@clermont.unicancer.fr; 14 Department of Pathology, University of Clermont Auvergne, INSERM U1240, 49 bd François Mitterrand, CS 60032, 63001 Clermont-Ferrand, France; 15 Europa Uomo, Leopoldstraat 34, 2000 Antwerp, Belgium; ken.mastris@btinternet.com; 16 AstraZeneca, Rua Humberto Madeira 7, 1800 Oeiras, Portugal; Carla.Pinto@astrazeneca.com; 17 Antwerp University Hospital, University of Antwerp, Wijlrijkstraat 10, 2650 Edegem, Belgium; jan.van.meerbeeck@uza.be; 18 F. Hoffmann-La Roche Ltd., Grenzacherstrasse 124, 4070 Basel, Switzerland; marlene.thomas@roche.com; 19 Eli Lilly and Company, Rue du Marquis 1, Markiesstraat, 1000 Brussels, Belgium; sonia.ujupan@lilly.com; 20 Department of Pathology, Hadassah Hebrew-University Medical Center, Hebrew University of Jerusalem, Kalman Ya’akov Man St, Jerusalem 91905, Israel; giladwv@gmail.com; 21 University Medical Center Hamburg-Eppendorf, Martinistraße 52, 20251 Hamburg, Germany; j.velthaus@uke.de; 22 Institut Gustave Roussy, 114 Rue Edouard Vaillant, 94805 Villejuif, France; fabrice.andre@gustaveroussy.fr

**Keywords:** clinical standardization, equitable reimbursement, European Alliance for Personalised Medicine, Europe’s Beating Cancer Plan, evidence generation, governance, molecularly guided treatment options, next-generation sequencing, stakeholder awareness and education, testing infrastructure

## Abstract

Next-generation sequencing (NGS) may enable more focused and highly personalized cancer treatment, with the National Comprehensive Cancer Network and European Society for Medical Oncology guidelines now recommending NGS for daily clinical practice for several tumor types. However, NGS implementation, and therefore patient access, varies across Europe; a multi-stakeholder collaboration is needed to establish the conditions required to improve this discrepancy. In that regard, we set up European Alliance for Personalised Medicine (EAPM)-led expert panels during the first half of 2021, including key stakeholders from across 10 European countries covering medical, economic, patient, industry, and governmental expertise. We describe the outcomes of these panels in order to define and explore the necessary conditions for NGS implementation into routine clinical care to enable patient access, identify specific challenges in achieving them, and make short- and long-term recommendations. The main challenges identified relate to the demand for NGS tests (governance, clinical standardization, and awareness and education) and supply of tests (equitable reimbursement, infrastructure for conducting and validating tests, and testing access driven by evidence generation). Recommendations made to resolve each of these challenges should aid multi-stakeholder collaboration between national and European initiatives, to complement, support, and mutually reinforce efforts to improve patient care.

## 1. Introduction

New possibilities for improved cancer care are on offer from an advanced technology already demonstrating its significance—next-generation sequencing (NGS). This refined testing has the potential to allow for more focused and highly personalized treatment [1]. Efficient use of NGS can deliver patient benefits by identifying treatments (known as molecularly guided treatment options (MGTOs)) that closely match genomic driver alterations, and this type of technology is already becoming widely used in routine clinical practice across the globe [2]; up until now, however, only a limited number of patients with cancer actually benefit from this approach [3,4] (see Box 1 for an overview of NGS).

Box 1 **Advanced Testing Technologies.**

Summary: Next-generation sequencing (NGS) enables rapid, affordable, and actionable information on individual tumors (known as molecular tumor profiles), so that clinical decisions, or molecularly guided treatment options (MGTOs), can be tailored to each patient.

NGS is a high-throughput technology that can allow integration of molecular tumor profiles into clinical decision-making as part of precision oncology [1]. NGS can be carried out using targeted gene panels, whole-exome sequencing, or whole-genome sequencing [5]. Comprehensive genomic profiling is an NGS approach that detects novel and known variants of the four main classes of genomic alterations, as well as genomic signatures, to provide prognostic, diagnostic, and predictive insights that inform research or treatment decisions for individual patients across all cancer types [6]. High-throughput technologies permit MGTOs that target particular molecular alterations in a patient’s tumor, along with their corresponding markers [1]. There are also a number of liquid- and tissue-based NGS panels for use in solid tumors [7].

NGS is a high-throughput technology that can allow integration of molecular tumor profiles into clinical decision-making as part of precision oncology [1]. NGS can be carried out using targeted gene panels, whole-exome sequencing, or whole-genome sequencing [5]. Comprehensive genomic profiling is an NGS approach that detects novel and known variants of the four main classes of genomic alterations, as well as genomic signatures, to provide prognostic, diagnostic, and predictive insights that inform research or treatment decisions for individual patients across all cancer types [6]. High-throughput technologies permit MGTOs that target particular molecular alterations in a patient’s tumor, along with their corresponding markers [1]. There are also a number of liquid- and tissue-based NGS panels for use in solid tumors [7].

**Figure 1:**
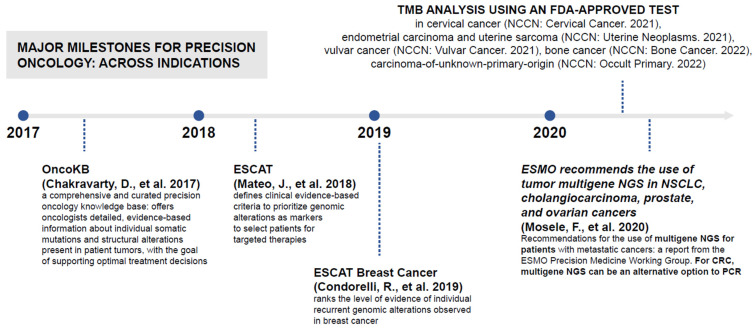
Evolution of support for genomic testing within guidelines. CRC, colorectal cancer; ESCAT, ESMO Scale for Clinical Actionability of molecular Targets; ESMO, European Society for Medical Oncology; FDA, Food and Drug Administration; NGS, next-generation sequencing; NSCLC, non-small cell lung cancer; PCR, polymerase chain reaction; TMB, tumor mutational burden.

Several MGTOs (see Box 1) are now approved for different tumor types by the US Food and Drug Administration (FDA), the European Medicines Agency (EMA), and other regulators, with many ongoing clinical development programs investigating MGTOs and genomic testing in these patients [1]. In addition, there are also companion diagnostic assays derived from liquid- and tissue-based NGS panels for use in solid tumors that have been approved by the FDA [7]. In addition to these regulatory approvals, the US National Comprehensive Cancer Network (NCCN) now recommends multigene panel testing for a number of other tumor types, also by using NGS [8,9,10,11,12,13,14]. This began with non-small cell lung cancer, but has since expanded to colon, prostate, breast, ovarian, carcinoma-of-unknown-primary-origin, and bone cancer [8,9,10,11,12,13,14]. Analysis of tumor mutational burden by an FDA-approved test is also recommended for cervical cancer, uterine neoplasms, vulvar cancer, carcinoma-of-unknown-primary-origin, and bone cancer [13,14,15,16,17]. Furthermore, the European Society for Medical Oncology (ESMO) Precision Medicine Working Group 2020 recommended NGS for daily clinical practice in several tumor types [18]. Additionally, some trials have demonstrated the feasibility of integrating NGS into routine clinical practice [19,20]. The growing support for genomic testing in guidelines is summarized in Figure 1. However, such support does not necessarily translate to equitable access to NGS within and across countries.

Across Europe, many patients with cancer will not benefit from NGS-driven approaches due to gaps in its implementation. Each country presently demonstrates different degrees of efficiency and deficiency in its approach to NGS, with widely varying practices in its use and access across Europe. Specifically, Germany, Denmark, Sweden, Finland, and the UK exhibit the highest uptake of NGS; countries with centralized systems that permit infrastructure investment (e.g., Denmark, Portugal, France, and the UK) demonstrate greater uptake than others [21]. Varying uptake and access also results from diverse reimbursement methods at a national and often regional level, where limited reimbursement of NGS restricts uptake [21]. For example, the UK, Belgium, Denmark, and the Netherlands mainly rely on national government-based funding [2]. Moreover, Estonia has used a variety of private, public, and government-based methods of reimbursement to fund the country’s ongoing personalized medicine project [22]. There are also variations in the content of NGS patient consent forms across Europe (e.g., in data retention policies) [23], and recommendations so far have been broad in nature, with little consensus [24], further impacting implementation of NGS. In addition, specialist advisory bodies, known as molecular tumor boards (MTBs) [25], have different constitutions and aims from country to country, and employ differing evidence scales when discussing the results of NGS (Table 1). Quality assurance provisions for NGS also vary, from mandatory in the UK and generally required in Belgium, Germany, and Slovenia, to absent in some Eastern European countries (e.g., Romania, Czech Republic, Hungary), potentially due to a lack of funding [21]. In addition to limiting the availability and access to NGS for many patients in Europe, this lack of European alignment also inhibits the emergence of best practice as well as cross-border understanding and sharing that could raise standards of care and advance the development of personalized medicine.

Achieving transformation of patient care with an NGS approach requires conditions for increased implementation to be met. This depends on collaboration between multiple stakeholders, including payers, policymakers, the medical and scientific community, and patient organizations, at both the national and the international level.

Implementation of NGS across Europe can be aided by linking national efforts, where care is delivered to in-need patients, and those at the European Union (EU) level. At the EU level, major policy initiatives in the health field are underway or in preparation, many of them offering direct or indirect pathways for implementation (e.g., Europe’s Beating Cancer Plan (BCP), a policy framework to establish resource allocation for and thereby improve implementation of personalized healthcare at the national level) [26]. Challenges to implementation of NGS at the national and EU levels are linked, since putting initiatives into effect is highly dependent on cooperation between these levels and could be hindered by shortfalls in interpretation or understanding.

**Figure 2:**
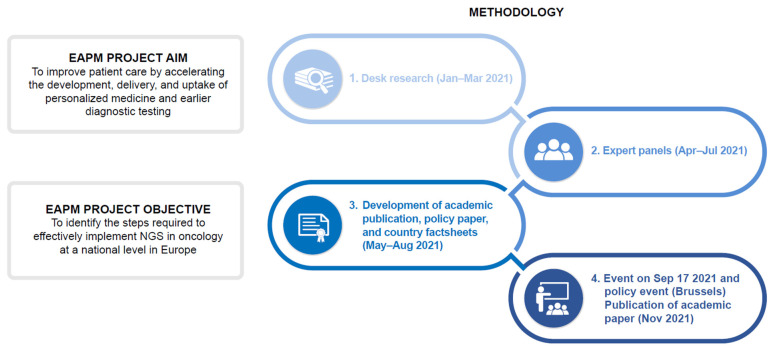
EAPM project overview. EAPM, European Alliance for Personalised Medicine; NGS, next-generation sequencing.

The European Alliance for Personalised Medicine (EAPM)-led expert panels, which took place with key stakeholders during the first half of 2021 (Figure 2), identified core challenges in implementing NGS into routine clinical cancer care nationally in 10 European countries (Belgium, France, Germany, Israel, Italy, Portugal, the Republic of Ireland, Slovenia, Switzerland, and the UK). Panel expertise included medical, economic, patient, and industry. We describe the outcomes of these expert panels to define and explore the necessary conditions for NGS implementation into routine clinical care, identify the specific challenges in achieving them, and make recommendations for effective implementation. Consensus on the most effective ways to make the necessary provisions for NGS is still incomplete, even within individual countries. Thus, a key recommendation is to examine where closer links between national and European initiatives could complement, support, and mutually reinforce efforts to improve patient care.

## 2. Core Challenges

The panels identified a number of critical challenges to efficient and widespread implementation of NGS for oncology across Europe. Mainly, as the complexity and vast amounts of data generated through advanced molecular profiling techniques such as NGS increases [27], the one-biomarker-one-therapy approach becomes less relevant, and the I-PREDICT trial has already demonstrated the feasibility of NGS-based, customized multi-drug regimens in patients with refractory malignancies [19]. Implementation of such an approach is challenging for many systems and is therefore the main roadblock for NGS implementation across Europe.

**Figure 3:**
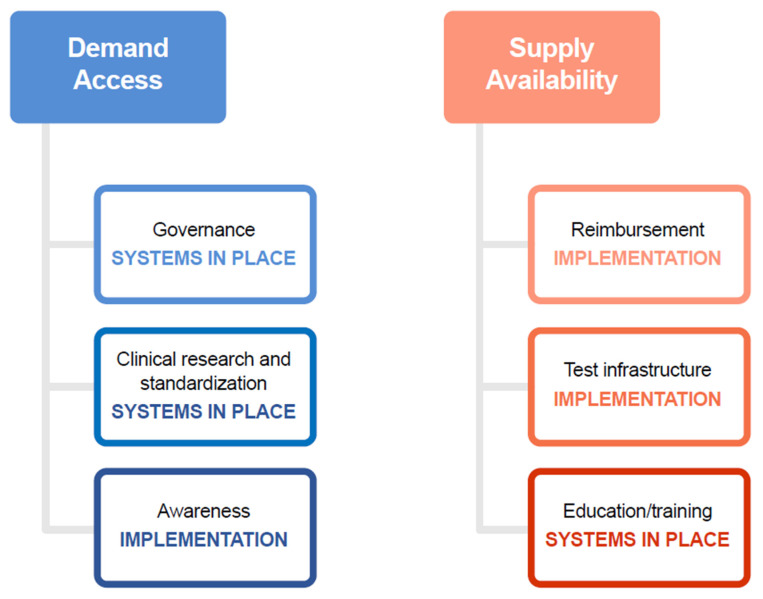
Overview of demand and supply challenges facing the implementation of NGS across Europe. NGS, next-generation sequencing.

More specifically, the main challenges relate to the demand for tests (which is influenced by governance, clinical standardization, and awareness and education) and supply of tests (influenced predominantly by equitable reimbursement, infrastructure for conducting and validating tests, and testing access driven by evidence generation; Figure 3). The panels also made recommendations for boosting NGS implementation, as detailed in Section 4.

### 2.1. Key Challenges to the Demand for Tests

#### 2.1.1. Governance

Governance relates to the level of coordination between public and private sectors involved in policymaking at a national and global level [28]. Key governance-related challenges identified during the expert panels included:

1. Insufficient harmonization of clinical infrastructures (e.g., data capture through electronic health records and limited laboratory and analytic services) due to the absence of national strategies for the implementation of personalized medicine approaches, confounded further by the absence of a pan-stakeholder group (i.e., physicians, patients, payers) well placed to advise on the implementation of NGS.
2. Lack of oversight in the implementation of policies and guidelines, as well as a lack of a regulatory framework to aid implementation. For instance, in France, implementation of ESMO recommendations currently varies between different institutions and laboratories, due to varying awareness of such guidelines.
3. A dynamic environment, with increasing numbers of innovative MGTOs and clinical trial designs, and greater volumes of genomic data from high-throughput technologies. Healthcare systems are often not ready to respond, and there are typically logistical challenges related to data management and sharing due to General Data Protection Regulation requirements and ever-increasing demands for data storage, and difficulties with data collection and harmonization.
4. A wide range of country-specific governance challenges that require resolution (summarized in Table S1).

#### 2.1.2. Clinical Standardization

Our expert panels identified multiple challenges related to clinical standardization, thereby impacting implementation of NGS into routine clinical cancer care:

1. Among clinicians, there are variations in the patterns of ordering large gene panels (>50 genes), due to lack of validation and standardization of in-house biomarkers and testing procedures, as well as varying application of International Organization for Standardization (ISO)-based accreditation (ISO 15189) [29] between hospitals. Standardization between hospitals may be difficult to achieve due to the complexity of NGS, but can be aided by the use of commercial NGS test providers that have achieved clinical validation for particular biomarkers [7].
2. Insufficient guidance on evidence requirements to demonstrate clinical utility of NGS.
3. Although some MGTOs have demonstrated clinical activity across multiple tumor types sharing the same molecular alteration (e.g., *NTRK* fusion, microsatellite instability, DNA mismatch repair-deficiencies [30,31]), leading to broad or even tumor-agnostic approvals, there remains insufficient understanding of the varying efficacy in different tumor types. For example, the poly(ADP-ribose) polymerase inhibitor olaparib leads to an improvement in progression-free survival of ~3 years in patients with *BRCA1/2*-altered ovarian tumors [32], compared with up to ~7 months for *BRCA1/2*-altered breast, pancreatic, or prostate cancer [33,34,35]. Furthermore, dabrafenib plus trametinib has demonstrated varying objective response rates of 61–68% between patients with previously untreated melanoma and non-small cell lung cancer [36,37].
4. Factors affecting choice of test type, such as cancer type, age, gender, high and low scoring of tumor mutational burden, and challenges related to quality assurance and reliability of assay results.
5. Limited implementation of NGS testing is also often due to several country-specific challenges, as identified in Table S2 [38,39].

#### 2.1.3. Awareness and Education

Stakeholder awareness of NGS varies throughout Europe. As identified during the expert panels, access to NGS can be limited by low awareness among physicians of the availability of biomarker tests and limited knowledge of referral pathways, particularly in rural centers. Physicians also show uncertainty when interpreting and using genomic data to guide treatment; a survey across 19 European countries found 39% of clinicians to be dissatisfied with the conditions of non-small cell lung cancer molecular testing in their country, citing difficulties with understanding results [40]. This is confounded by the differing education of physicians across Europe. Awareness of NGS is also limited among patients; one survey found that 70% of patients with cancer reported not having received explanations of the importance of molecular testing [41]. Country-specific challenges and recommendations related to stakeholder awareness and education are summarized in Table S3.

### 2.2. Key Challenges to the Supply of Tests

#### 2.2.1. Reimbursement

There are challenges to equitable reimbursement and subsequent access to NGS across Europe, as highlighted in our expert panel discussions and summarized in Table S4 [42]. Key general challenges include:

1. Lack of value assessment processes for diagnostics, including NGS, in many countries and a generalized lack of formal pathways for reimbursement. There is also a lack of pathways for reimbursement that are time-bound and flexible and that accommodate the unique characteristics of the advanced diagnostic [43].
2. Timing to access for advanced diagnostics is currently a major barrier to adoption.
3. Absence of a widely accepted health technology assessment (HTA) framework for demonstrating the value of diagnostic methods, including NGS, as well as other health technologies, to improve access to targeted therapies.
4. Inconsistency across countries in whether NGS should be evaluated and reimbursed at the generic level or for the class of test (e.g., based on the number of base pairs sequenced/size of the gene panel). Inconsistent evaluations are also a result of a lack of evidence frameworks for NGS, including broadly agreed-upon standards to demonstrate clinical utility, mitigate uncertainty, and secure public/private reimbursement [44].
5. In some countries, lack of reimbursement codes for NGS or insufficient coverage for laboratories to conduct NGS.
6. Insufficient laboratory/hospital budgets to cover growing volumes of NGS testing, and siloed budgets between diagnostics and hospitals, leading to infrastructural challenges (e.g., obtaining an appropriately trained workforce, data sharing and data storage infrastructure capabilities, and digital health recording within healthcare systems) [43]. Such budget insufficiency is also seen more broadly for non-academic stakeholders co-investing in a public–private partnership, e.g., via an innovation fund.

The novelty of advanced diagnostic tests is not matched by current value assessment frameworks, which omit key aspects of the potential value of diagnostic tests and apply the same, often narrow, value framework used for medicines [45]. Furthermore, frameworks differ across Europe, with some countries placing more of an emphasis on clinical outcomes, and others on cost-effectiveness [46]. NGS test manufacturers must replicate evidence in every country in Europe to demonstrate that NGS can be implemented. These challenges limit equitable reimbursement and timely access to NGS testing across Europe. Current value assessment frameworks also often fail to recognize the potential value of advanced diagnostic tests where MGTOs have demonstrated clinical benefit across several different solid tumor types sharing the same molecular alteration. Even when the EMA has granted tumor-agnostic regulatory approvals, subsequent reimbursement of these therapies in European countries is not always followed by the timely reimbursement of the appropriate advanced diagnostic, as elucidated in our expert panel discussions. For example, in Belgium, although crizotinib was approved in 2013, the approval for reimbursement of the companion diagnostic test for identification of *ALK* fusions occurred only in 2019 [47]. Overall, therapies and advanced diagnostics have different reimbursement procedures (e.g., time limits for reimbursement, decision-making bodies, value frameworks), which vary across Europe [47].

Such challenges delay time to access advanced diagnostics, with potentially 5–10-year delays from initial launch (e.g., Oncotype Dx was only reimbursed by the National Health Service [NHS] in the UK in 2015, 9 years after reimbursement in the US) [43]. To ensure more efficient patient access, positive diagnostic appraisals should lead to automatic reimbursement in a set time frame, and payers should be more willing to utilize innovate reimbursement schemes to demonstrate value [43].

#### 2.2.2. Infrastructure

Our expert panel discussions identified many infrastructure-related challenges, including:

1. Differences in centralized/decentralized testing infrastructure requirements.
2. Lack of alignment of availability of bioinformatics technology (e.g., data management, analysis, and storage) across Europe.
3. Limited knowledge and capacity for interpreting NGS reports.
4. Variable and inconsistent methods of ordering biomarker testing.
5. Insufficient funding for NGS.
6. Insufficient efforts to resource and train a specialized workforce.
7. Lack of harmonization across datasets, limiting application of artificial intelligence.
8. Varying levels of centralization and infrastructure capacity across Europe, leading to several country-specific recommendations (Table S5).

#### 2.2.3. Access to Tests Linked to Evidence Generation and Supporting Infrastructure

As advanced diagnostics progressively identify new molecular subtypes, there is a concomitant reduction in the number of patients within each molecular subtype. Overall, the need to recruit smaller and more precisely defined populations renders traditional clinical trial designs for MGTOs costly and time-consuming. Innovative clinical trial designs, such as enrichment, basket, umbrella, and adaptive trials (e.g., MINDACT [NCT00433589], TAPUR [NCT02693535], PROFILER 02 [NCT03163732], Lung-MAP [NCT02154490], GBM AGILE [NCT03970447]) [48,49], as well as combinations of these (e.g., DRUP [NCT02925234]) [20], are therefore important in this context.

Nonetheless, precision oncology clinical trials are challenged by the high screening numbers required [50], which may be compounded by recruitment hesitancy. This may be due to recent results from pan-cancer clinical trials demonstrating limited clinical utility of NGS-based molecular therapy (although such trials included heavily pretreated patients with advanced disease and were limited by long processing times of genomic results and low molecular matching rates) [3,4]. Furthermore, traditional trials usually focus on a single genomic alteration with narrow patient eligibility criteria; therefore, it is unclear how applicable results from a clinical trial are to a real-world setting [51,52].

Real-world data (RWD)-based studies, which use data related to a patient’s health status or the delivery of healthcare in routine clinical care to generate real-world evidence (RWE) [53], are therefore vital in complementing clinical trials to aid evidence generation and extend NGS to both preventative and curative roles [45]. However, our expert panel discussions identified multiple challenges in limiting use of RWE in precision oncology, including:

1. Lack of national registries.
2. Data standardization and harmonization.
3. Data protection legislation.
4. Lack of European-level regulatory guidance for institutions conducting RWD-based studies that sufficiently address concerns from payers or regulatory bodies, including RWD quality and comprehensiveness, data standardization, methodological challenges and lack of transparency, failure to collect or prioritize data relevant to coverage decision-making (health resource utilization), and small sample sizes [46,54,55].
5. Biased RWE due to connections with groups assessing reimbursement.
6. Incorporation of RWE into the value assessment process, which is critical for securing reimbursement and enabling an RWE-based learning healthcare system to continually study, learn, and improve precision oncology.
7. Various country-specific challenges (Table S6).

## 3. Relevant Recent and Upcoming NGS-Related Initiatives

Europe, and its individual Member States, are at a pivotal moment in the evolution of healthcare policy, driven in part by a determination to recover from the recent COVID-19 pandemic and to prepare for future crises [56]. It is also driven by an ever-sharper awareness of the underlying crisis in healthcare, where demographics and chronic disease are on a collision course with healthcare financing, unless there are radical changes to the current approach and better use is made of innovative methods. In this context, the importance of NGS-driven, high-tech screening and diagnostics have been given new prominence by the exigencies of the COVID-19 pandemic; the continually rising burden of cancer in Europe [57] has also lent new significance to technologies that can deliver more selective and effective treatments, with benefits for health budgets as well as for patients. For example, in advanced non-small cell lung cancer, NGS has been shown to have a modest budget impact for payers, with the potential to better enable selection for targeted therapy and clinical trial enrollment [58,59,60,61].

In recent years, there has been a surge of health-related policy and practice initiatives both from individual countries and from EU institutions, fortuitously offering scope for advancing understanding and development of NGS [62]. Some of these initiatives are COVID-19-independent and predate the pandemic; others arose as a direct response to the sudden demonstration of the effect that a virus can have on public health and healthcare systems. Several countries have also established MTBs to help exploit the potential of NGS and precision oncology, offering the possibility of harmonizing the interpretation of NGS results and the subsequent translation into clinical action and benefit, particularly for patients with rare tumors or those for whom standard-of-care therapy has been unsuccessful (Table 1) [25].

As highlighted in our expert panel discussions and summarized in Tables S1–S6 and Table among the list of national initiatives, various country-specific advances have been made. For instance, the process of clinical laboratory standardization is underway in Belgium, which has set up a specific annual budget for NGS. Clinical standardization is also under discussion in Germany, where there are national personalized medicine centers working to develop national recommendations for NGS-related RWE collection and standardization. Germany also has included outcome data in a centralized data pool accessible by all university hospitals. In Poland, oncologists with expertise in molecular diagnostics are preparing recommendations for NGS implementation for publication. Portugal is also attempting to develop national registries to improve evidence generation. In Italy, meetings among oncologists have been arranged to improve knowledge and application of NGS [63], and the Netherlands have committees that determine diagnostics and treatment plans for cancer. In the Republic of Ireland, a national cancer genomics strategy has begun to aid testing harmonization. Genomic Medicine Sweden is a national genomic initiative aiming for implementation of large-scale sequencing techniques and genomics in healthcare for patients with rare diseases and cancer [64]. In the UK, NGS is now accessible to a limited extent via the opening of seven regional genomic hubs by NHS England [65], although, as of yet, there is no established strategy for the devolved nations in the UK.

However, such local initiatives remain disconnected from EU-wide standards, and although the ESMO Precision Medicine Working Group 2020 now recommends NGS for daily clinical practice in several tumor types (advanced non-squamous non-small cell lung cancer, prostate cancer, ovarian cancer, and cholangiocarcinoma) [18], this has not translated into equitable guidelines across Europe. For example, although Swedish non-small cell lung cancer guidelines recommend NGS-based genomic testing for *EGFR*, *KRAS*, *BRAF*, *ALK*, *NTRK*, and *ROS1*, guidelines in Norway only include *EGFR*, *ALK*, and *ROS1* [66]. In Spain, guidelines do recommend molecular testing of *EGFR*, *ALK*, *ROS1*, and *BRAF1*, but only suggest additional testing of genes such as *KRAS* and *NTRK* if previous biomarker testing yields negative results [67]. Moreover, although these guidelines advise NGS-based profiling, they also acknowledge the value of other techniques, such as fluorescent in situ hybridization and immunohistochemistry [67]. In breast cancer, Spanish guidelines suggest that an optimal gene panel should detect *AKT1*, *PIK3CA*, *PTEN*, and *ESR1* mutations and *FGFR1* amplification, in addition to studying estrogen receptor, progesterone receptor, *HER2*, and *BRCA1/2*, although NGS is still only considered a research tool [68]. In Germany, only *BRCA1/2*, *PIK3CA*, *NTRK*, and MSI have an AGO (Arbeitsgemeinschaft Gynäkologische Onkologie) recommendation of + or ++ [69]. In Sweden, the guidelines are much less clear; although they acknowledge the treatment predictive value of *HER2*, *BRCA*, *ESR1*, and *PIK3CA* and the importance of broad genomic profiling, no formal recommendations have been made [70].

At the European level, prior to the COVID-19 pandemic, there were attempts to adopt a more proactive stance on health in the face of well-recognized challenges, from cancer care and data collection to closer collaboration. There were proposals for creating a European Health Union [71], although the pandemic precipitated specific initiatives including joint procurement of medicines and boosting of Europe’s pharmaceutical capacity. Some of the principal examples of these diverse initiatives, and their relevance to more efficient use of NGS, are indicated in this section.

The European Commission’s ambition to build an overarching European Health Union is explicitly geared toward improving prevention, treatment, and aftercare “for diseases such as cancer”, and, following the COVID-19 pandemic, has also been extended to strengthening the EMA to provide stronger surveillance, scientific analysis, and guidance [71]. It includes plans to modernize Europe’s regulatory framework for pharmaceutical companies to support “research and technologies that reach patients” [71].

Conceived in 2018, Europe’s BCP was launched in 2021; it emphasizes early detection, diagnosis, and treatment to improve patient outcomes, underlines the urgency for equitable access to innovation in diagnostic procedures, personalized cancer treatment, and data management, and identifies access to precision oncology (e.g., advanced diagnostics) as a relevant topic [26,46]. Europe’s BCP, as a policy framework, has recognized molecular diagnostics as highly relevant in cancer treatment pathways, from guidance on optimal treatment to monitoring of its effectiveness, in order to improve patient access and budget for ongoing research and development [72]. The “Cancer Diagnostic and Treatment for All” initiative under Europe’s BCP, to be launched by the end of 2021, aims specifically to improve access to innovative cancer diagnostic techniques and treatments, including the use of NGS technology, and will encourage broader access to precision oncology [26,46]. The parallel EU Mission on Cancer includes plans for a “European Initiative to Understand Cancer” in order to identify individuals at high risk from common cancers, which could promote personalized approaches to cancer treatment and early detection, as part of the Horizon Europe framework beginning in 2021 [26]. Europe’s BCP also includes a flagship registry initiative, aiming to establish a “Cancer Inequalities Registry” in 2021 that will identify trends, disparities, and inequalities in cancer treatment within Member States and regions, and explore areas of action to guide investment and interventions at European, national, and regional levels [26]. The creation of a European Health Data Space is another of Europe’s BCP’s priorities for the next 3 years, aiming to promote better exchange and access to health data in electronic health records. With strong data governance, data quality rules, and a strong infrastructure for interoperability, it will assist Member States in sharing health data across EU borders for public health, treatment, research, and innovation [26]. Moreover, as part of Europe’s BCP, the 1+ Million Genomes initiative of the European Commission brings together 22 EU countries, plus the UK and Norway, with the goal of having at least one million sequenced genomes accessible in the EU by 2022. An EU Cancer Plan Implementation Group will align actions and policies across the European Commission and other EU institutions [26]. Finally, within Europe’s BCP, the harmonization of Comprehensive Cancer Center standards will be discussed, which will hopefully improve delivery of care by aiding more equitable implementation of standard-of-care.

A European Parliament report on Europe’s BCP drafted in mid-2021 highlighted the importance of patient referral to diagnostic tests and oncology specialists via general practitioners, pediatricians, and primary care professionals, and urged promotion by the European Commission and Member States. The draft report also specifically spotlighted the merits of NGS for generating quick and efficient genetic profiles of tumor cells, enabling researchers and clinicians to share cancer profiles as well as to apply the same or similar diagnostic and therapeutic approaches to patients with comparable cancer profiles. It calls for a charter of rights for patients with cancer, defining their entitlement everywhere in the EU to prevention, initial diagnosis, and treatment. HTAs will also benefit from greater coordination among EU Member States, with the adoption in mid-2021 of a regulation that the European Parliament Cancer Committee welcomes as “harmonizing access to innovative cancer diagnosis and treatments.” Over the coming months and years, as the details of the EU HTA implementation are elucidated, there will be scope for advancing recognition of the importance of NGS.

The EU In Vitro Diagnostic Regulation (IVDR), applicable from May 2022, will require all manufacturers of in vitro diagnostic tests to obtain certification to distribute and sell their products within the EU, thus offering greater standardization of laboratory testing to ensure patient safety [73]. IVDR also provides greater opportunity for public–private partnerships [43]. Impact of the IVDR and actions required to prepare for this new regulation are discussed in Section 4.2.

The European Health Data & Evidence Network (EHDEN) is developing a federated network of real-world clinical data sources from health records across Europe, all standardized and harmonized to a common data model and with data privacy by design [74].

The ESMO 2020 guidelines, with recommendations for routine use of NGS in a number of common cancers, are a valuable response to the absence of recommendations from scientific societies regarding the use of NGS in oncology practice [18]. They also recommend selective use, including of large multigene panels, in less common cancers. The guidelines further support the development of multigene sequencing in clinical research centers as a tool to screen patients eligible for clinical trials, accelerate drug development, and prospectively capture data to further inform how to optimize the use of this technology. In addition, the guidelines note that economic evaluations, alongside clinical trials, should be implemented to foster evidence generation in this field [18]. Such cost-effectiveness evaluations have been performed previously [75,76,77], although they vary in their costing methods and in the cancer types studied, which must be standardized.

These initiatives, many of them with associated specific funding possibilities, take their place alongside a range of more general funding possibilities for NGS development through EU programs aimed at research, digitalization, regional development, and social care.

## 4. Detailed Recommendations

The EAPM-led expert panels identified, in addition to challenges to increased implementation of NGS uptake, recommendations, with implications for action at both the national and the European levels. As in Section 1 (on the challenges identified), we developed a similar set of recommendations relating to the demand for and the supply of tests (Figure 3).

### 4.1. Governance

**Figure 4:**
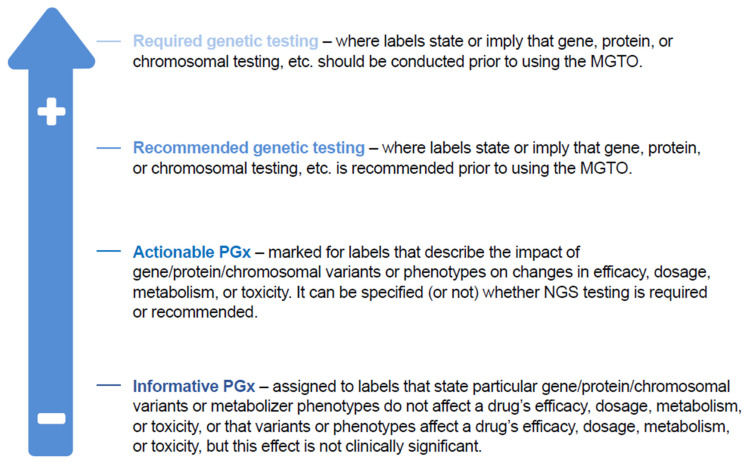
PharmGKB classification of PGx information associated with specific gene–drug combination. MGTO, molecularly guided treatment option; PGx, pharmacogenomics. Adapted from Kim J. A., et al., 2021 [81].

1. Evidence generation: Demonstration of the clinical utility of NGS to patients, both at baseline and at tumor progression, is critical in supporting national initiatives and harmonized implementation of policies and guidelines. As NGS typically succeeds on a case-by-case basis, this would allow for selection of the most appropriate patients to receive NGS. Traditional randomized clinical trials may not easily generate such evidence but can be aided through innovative clinical trial designs and RWD-based studies [45].
2. Up-to-date strategies: Alignment of national precision medicine strategies across Europe with the ever-changing environment of NGS and innovative treatment options. This can avert the risk of local resistance that inhibits the implementation of European NGS policies, such as Europe’s BCP or ESMO recommendations. Divergent interpretations in France of the ESMO recommendations highlight the need for more formal recommendations from the country’s National Cancer Institute.
3. Maximal European cooperation: European-level cooperation is required to discuss challenges of patient access to NGS and to ensure equal implementation across the Member States of the European Commission’s 1+ Million Genomes initiative, as well as other initiatives, including EHDEN and the European Health Data Space [26,74].
4. NGS standardization: National strategies must aim to: regulate NGS standardization and quality; clinical interpretation of genomic variants (based on, e.g., the ESMO Scale for Clinical Actionability of molecular Targets (ESCAT)) [78,79] and access to evolving treatment options; methods of measuring tumor mutational burden; efficient collection and use of tumor samples; privacy and ethics in relation to data sharing and participation in international clinical databases; data interoperability for use; and the tumor types that incorporate RNA (for gene fusions and, in the near future, gene expressions), circulating tumor DNA, or epigenetic analysis as part of testing. This is necessary for consistency between laboratories performing genomic testing, including consistency regarding quality, the test/platform used, and the interpretation of results, particularly in those countries without laboratory accreditation. Patients and patient advocates should be involved in the discussion of privacy and ethics to advance the application of robust patient consent procedures in all Member States, with clear guidance on who should and can obtain consent, and educational initiatives to equip clinicians accordingly [80].
5. Clear classification: National strategies should classify the use of NGS testing in the context of clinical decision-making and delivery of care. A US method exists to classify pharmacogenomic information from a particular gene–drug combination as clinically actionable (PharmGKB, Stanford, CA, USA; Figure 4) [81]; however, translating this classification system to routine clinical practice would require a clear definition of the aim of NGS testing (i.e., diagnosis, assessing immediate treatments decisions, identifying actionable alterations), clear delineation of the difference between genomic insights based on clinical validation (i.e., companion diagnostics as required by the new IVDR or based on Software as a Medical Device) and insights based on research-use-only techniques, and attention to the importance of the pharmacogenomic information that can be vital for disease prevention and for understanding potential drug toxicities. Consistent interpretation of NGS results depends on standardized MTB evidence scales, which are reliant on validated bioinformatics analysis pathways, variant calling, and pipeline analysis. OncoKB is a knowledge base offering evidence-based information about individual somatic mutations and structural alterations present in patient tumors [82]; it has an important role in improving reproducibility and accuracy of MTB findings. ESCAT also helps to rank targets for precision oncology based on clinical evidence of their utility and to prioritize targets for clinical use [78,79].
6. Clinically useful MTBs: MTBs are critical to accommodate the ever-evolving environment of NGS and precision oncology, to harmonize interpretation of NGS results and thus aid translation into clinical action and benefit [25]. To maximize its clinical utility, it is necessary to clearly define the purpose of an MTB, to establish guidelines on minimal membership, operational requirements, and measures of quality, and to secure global harmonization in the sharing of clinical data experiences between institutions. It is also critical to accommodate virtual MTBs in a time- and location-independent manner between cancer care teams comprising experts across several disciplines. MTBs must be reactive and adaptive to recent clinical trial results and capture patient outcomes to validate clinical decision-making as well as support a learning healthcare system. Several further country-specific recommendations are summarized in Table 1.

### 4.2. Clinical Standardization

1. Clear, up-to-date, and dynamic guidance: Guidance describing when and where NGS should be performed, including the genes and patient populations to be analyzed, minimum standards for testing to ensure high-quality NGS, and limiting NGS to laboratories with ISO-based accreditation (according to the ISO 15189 standards) [29], as a basis for establishing an external quality assessment system.
2. NGS workflow standardization and optimization: Standardization and tracking of the entire NGS workflow, including the pre-analytical (i.e., tissue quality, tumor content quantification, DNA fragmentation, and library preparation), DNA sequencing, coverage, and data-analysis steps. Standardization is also essential to avoid outliers when using NGS for precision-oncology-based research. In addition, optimization of the NGS workflow, particularly in relation to turnaround time, is key. Europe’s BCP offers a model aimed at ensuring high-quality innovative treatments and diagnostics, with an EU Network aiming to link recognized National Comprehensive Cancer Centers in every member state by 2025 and guarantee access to such centers for 90% of eligible patients by 2030 [26]. Such centers should involve external stakeholders to promote multicenter collaboration.
3. European collaboration to address the IVDR: In-house laboratory in vitro diagnostic tests are essential to ensure broad coverage of healthcare in many diagnostic fields and will be impacted by IVDR, which limits their use to situations in which appropriate certified in vitro diagnostic tests are not available [73]. Diagnostic processes, for instance to validate individual genomic alterations, may be complicated by the IVDR. Moreover, many current NGS tests are not performed according to IVDR requirements, which will lead to a steep increase in the number of tests requiring certification [73]. Compliance with guidelines may require test revalidation and significant replacement of technology, but, given limited funding in some countries, the result may be an increase in laboratory reliance on commercial providers leading to concentration among laboratories. In addition, in the dynamic and fast-moving fields of precision oncology and molecular pathology, laboratory-developed tests play a central role, as they often drive diagnostic innovation in collaboration with industry when no approved testing options exist. Nonetheless, the IVDR, established in 2017, is key to ensuring high-quality, standardized, and high-throughput testing, and it aims to guarantee safe and effective use of the corresponding medicinal product [83]. This may address many of the challenges detailed above, and laboratories must prepare for this legislation. In that regard, laboratories should appoint a small team dedicated to ensuring compliance with IVDR regulations and prepare an inventory of all currently implemented commercially available and laboratory-developed tests. Furthermore, laboratories must engage in European collaboration as part of multicenter studies to generate data related to laboratory-developed tests and obtain appropriate certification, and consult published IVDR guidance documents from the European Commission (although some of these documents are still pending) [73].

### 4.3. Awareness and Education

1. Physician, molecular pathologist, and bioinformatician education: Education should equip physicians, molecular pathologists, and bioinformaticians to order, interpret, and use genomic evidence to guide treatment, from students and trainees (e.g., updated undergraduate genomics curriculum) to consultants and associate specialists, providing advice at clinical genetics services and genomic laboratory hubs [80]. MTBs and clinical-decision-support systems should be used to assist this process. It is important that regular, objective meetings designed for postgraduate training be organized and that they be able to indicate clear arrangements and plans for infrastructure or reimbursement.
2. Involvement of patient advisory groups: With the assistance of patient advisory groups, patients’ preferences should be considered throughout the regulatory (e.g., value assessments) and scientific processes, and it is key to manage patients’ expectations of NGS. For example, patient organizations in the Netherlands are requesting the release of NGS reports to both patient and clinician, which will help to raise awareness of NGS and precision oncology across all tumor types.
3. Broader national initiatives: There should be wider use of national initiatives, such as the Health Education England’s Genomics Education Programme and the NHS’s Genomics Clinical Reference Group (Box 2), which serve as examples of connecting genomic data sources and combining data with clinical RWD. There are no equivalents in Portugal and Italy, and such national initiatives could also benefit Germany’s multiple ongoing initiatives (e.g., German Cancer Aid). As part of integrity and harmonization, the UK initiatives should be used as an approach to connect genomic data sources and combine data with clinical RWD in a centralized, aggregated manner across Europe.
4. Framework from Europe’s BCP: Europe’s BCP could provide a framework for these recommendations in its recruitment of social workers, teachers, and nurses to improve public health literacy on cancer risks and determinants, as well as to educate patients on healthy behavior and on how they can live well following cancer treatment. Europe’s BCP also aims to use training and continuous education of healthcare professionals, including on digital skills, artificial intelligence, genomics, and personalized medicine, to build a stronger multidisciplinary cancer workforce. This will help Member States to address skills gaps and equip their health workforce with personnel trained in cancer prevention, early detection, diagnosis, treatment, rehabilitation, and survivorship. Furthermore, as part of the “Partnership on Personalised Medicine” (due to be set up in 2023), Europe’s BCP will establish and support priorities for personalized-medicine-related research and education and provide guidance on the implementation of personalized medicine approaches into routine clinical care. Moreover, the “European Initiative to Understand Cancer” aims to increase understanding of cancer development in order to identify individuals at high risk of developing cancer and therefore better facilitate personalized approaches to cancer prevention and care. Finally, Europe’s BCP’s Comprehensive Cancer Centers, planned to be established by 2025, will also play a key role in the above recommendations; for instance, they will facilitate diagnostic- and treatment-related training, research, and clinical trials across the EU, helping to improve patients’ access to these aspects of personalized medicine [26].

Box 2 **Awareness: United Kingdom Best Practice.**

Genomics Education Programme [84]: Exists to deliver and advise on learning and development opportunities to prepare current and future National Health Service (NHS) professionals to make the best use of genomics in their practice. The program aims to prime the NHS to deliver the new England-wide Genomic Medicine Service, support the 100,000 Genomes Project, facilitate genomic education opportunities for NHS professionals, and maintain and enhance collaborations to keep the UK at the forefront of genomics.

Genomics Clinical Reference Group [85]: Aims to support the implementation of the NHS Genomic Medicine Service, with the following objectives:

1. To advise on implementation of NHS long-term commitments and development of the NHS Genome Medicine Service.
2. To facilitate an annual review of the National Genomic Test Directory.
3. To enhance awareness and implementation of genomics across all clinical specialties.
4. To drive improvements in personalized medicine.
5. To advise on, review, and develop genomics guidance and testing specifications.

### 4.4. Reimbursement

Two main pathways for reimbursement of targeted therapies/testing exist, including national access body decisions (e.g., recommendations for use of larotrectinib and entrectinib as part of the Cancer Drugs Fund from the UK National Institute for Health and Care Excellence) [86] and early access schemes to provide patients with life-threatening or debilitating conditions access to medicines that do not yet have a marketing authorization (e.g., early access in Italy for selpercatinib). However, a major additional pathway in Germany is through insurance companies, which have contracts with large, specialized centers that provide innovative testing as part of precision oncology. In Germany, manufacturers provide commercial NGS services through standard reimbursement codes and rates; in the outpatient setting, companion diagnostics are reimbursed by statutory health insurance under Einheitlicher Bewertungsmaßstab (extra budget until end of 2023; code includes not only the test itself but everything necessary to conduct it), whereas in the inpatient setting, code billing by German diagnosis-related groups (fee-per-case system) is required [87]. Moreover, an expert panel member from France has suggested that patient pressure on the Ministry of Social Affairs and Health has proved more effective in improving reimbursement than pressure from physicians. Therefore, due to the varying reimbursement pathways at a national level, several country-specific recommendations for reimbursement were made during the expert panel discussions (Table S4).

Some general recommendations were also made:

1. Standardized, comprehensive value assessment frameworks: Value assessment frameworks dedicated to advanced diagnostics must be standardized across Europe to incorporate the perspective of multiple stakeholders on a wider range of factors than is currently the case. These include: short- and long-term clinical utility as NGS moves away from the one-biomarker-one-therapy approach, patient preferences and utility, uncertainties in data, cost-effectiveness and cost-utility analyses that consider the full economic impact of all components of the intervention, ethical and legal implications, and equity-of-access and uptake requirements [88,89,90]. The frameworks should also enable use of innovative clinical trial designs and RWD-based studies, and take into account other patient perspectives, including quality of life, work productivity, caregiver and family burden, unmet need, burden of illness, and patient-reported outcome and experience measures. To more closely link reimbursement of the therapy and the diagnostic test, value assessment frameworks should incorporate the value of both [88]. Finally, value assessment frameworks should recognize the value of NGS in providing hereditary, diagnostic, prognostic, and treatment-response-related genomic information [91], or of using NGS for clinical trial allocation as a part of a patient’s current and future care plan.
2. Value assessment framework-guided evidence generation: Standardization of fast and efficient reimbursement of advanced diagnostics across Europe, and consequent harmonization of access, depends on value assessment framework-guided evidence generation demonstrating the clinical utility of NGS through innovative clinical trial designs and RWD-based studies assessing the safety and efficacy of NGS-based MGTOs. Evidence generation also requires guidance from regulatory bodies to ensure that requirements are met. In this way, improved evidence generation should convince payers of the value of these technologies in informing clinical decision-making. For example, NGS can offer better turnaround times than simultaneous hotspot panel testing, and thus time-to-therapy initiation, so that initial high costs may be offset by overall longer-term cost-effectiveness and the improved survival benefits associated with precision oncology [75,92]. There is scope for strengthening the case through increased access to MGTOs and improved incremental benefit compared with current treatments [76].

### 4.5. Infrastructure

1. Aligned methodologies across Europe: Harmonization of methodologies and analysis between laboratories at a national and European level will make it more feasible to tackle the huge volumes of data generated by NGS, which require significant infrastructure for secure data storage, analysis, and interpretation. European guidelines, analogous to those already in force in the US [93], are needed to promote alignment. Existing infrastructure should be leveraged with national genomics policy initiatives.
2. Use of artificial intelligence: Infrastructure for the bioinformatics pipeline should incorporate artificial intelligence, which has already been demonstrated as improving NGS-based diagnostics in accuracy of variant identification [94], distinguishing between benign and pathogenic variants [95], user-friendliness of electronic health record systems [96], and linking multiple types of “omics” (e.g., genomics, transcriptomics, proteomics) and clinical data to better inform treatment decisions [97]. Artificial intelligence may also aid secure, harmonized data storage in line with privacy laws [98], and datasets to which artificial intelligence algorithms could be applied must be standardized and appropriately prepared.
3. National infrastructural initiatives: Projects critical for patient diagnosis and management must be developed, for example with the goal of the UK’s Genomic Medicine Service (within the NHS) of sequencing 500,000 whole genomes by 2023/2024 [62]. Such initiatives depend on a national network of genomic laboratory hubs, with the relevant NGS provision, data, and informatics infrastructure. This is easier in countries with a formalized healthcare service (e.g., the UK, the Netherlands [99], and Estonia [100]) than in countries with insufficient funding/capacity (e.g., Italy and France) or a federal structure (e.g., Germany). All national initiatives should incorporate a common language between institutions/laboratories to facilitate data integration and sharing, as well as elements of data protection.
4. Centralized, standardized centers for NGS excellence: Solutions must be found to fulfil the size, complexity, and cost of the information technology infrastructure required for NGS platforms, and the bioinformatic and molecular pathology expertise required to interpret NGS data. A promising option is to establish centralized, standardized centers of excellence in each country for MTBs and molecular oncology, capable of issuing standardized, clear recommendations that are backed by professional laboratories performing high-quality, end-to-end NGS services. Such a centralized approach may be particularly effective in small countries, such as Slovenia [101]. As the cost of NGS platforms decreases in the future and the technology becomes simpler to use [30,102,103], a decentralized model may become more appropriate, with several cancer centers carrying out sequencing and sending the results to a central bioinformatics core; such a model requires consistent guidelines to ensure standardization of preanalytical sequencing factors [104], and may increase turnaround time [105]. It is important to note, however, that the relative degree of (de)centralization appropriate will vary between countries, depending on specific factors (e.g., technology/personnel available).
5. In-house and commercial NGS platforms: In-house platforms for NGS often offer reduced costs [106], increased availability of raw data and DNA/RNA left for extraction, and opportunities for training of medical oncologists and pathologists. Although in-house platforms may also demonstrate improved turnaround times compared to commercial tests [107,108], this is highly dependent on the number of samples for in-house platforms (turnaround time for commercial platforms, which may offer increased sample numbers, is consistent) [107]. Nonetheless, commercial tests may enable incorporation of recently approved biomarkers more easily [109], and in-house platforms, although feasible for the wet/bench portion of NGS, are more challenging for smaller institutions when considering bioinformatics, clinical informatics, and data analytics, versus commercial platforms [110]. Careful selection of the most appropriate NGS platform and expertise is critical, and depends on prior assessment of laboratory workflow, structure, and NGS requirements and aims [106,111]. Careful review of current laboratory personnel, with additional training if appropriate, is necessary to ensure availability of sufficient expertise [111]. Regulatory requirements are also key to ensure that patients are not at risk of misdiagnosis or of missing out on MGTOs. Whichever platform is chosen, it must comply with the incoming IVDR.
6. Public–private partnerships: The involvement of local laboratories, public–private partnerships, and connectivity between local hospitals can assist broad knowledge generation and the translation of advanced diagnostics and innovative therapies in precision oncology to the community setting. Public–private partnerships lead to better communication and data sharing between the industry, genomic centers, and clinics [43].

### 4.6. Access and RWE

RWD-based studies provide indispensable complementary data to clinical trials to aid evidence generation, permitting the extension of NGS to both preventive and curative roles. RWE recommendations from the expert panels include:

1. Standardized, global, and centralized registries: As lack of data standardization can limit access to and interpretation of RWE across Europe, networks of standardized, global (or at least pan-European), and centralized multi-stakeholder registries collecting data related to diagnosis, treatments, and outcome, must be created. To permit data interoperability, pooling from disparate resources, and meaningfully comparable and reproducible results, all data variables collected should comply with the FAIR (Findable, Accessible, Interoperable and Reusable) Principles on data management [112] and be harmonized according to global data standards, terminologies, and models. Patient-reported outcomes will also be needed to enable adjustment for potential RWE bias. The federated network of the EHDEN [74] and the Cancer Inequalities Registry planned for 2021 as part of Europe’s BCP [26] offer potential platforms that could be usefully exploited. Such platforms may aid the governance-related infrastructural challenges detailed earlier.
2. Consensus guidelines for RWE: Pan-European regulation could give payers, regulators, and industry clearer guidance on the feasibility and implementation of RWE in clinical practice. European consensus guidelines could help to standardize RWD-based studies by providing guidance on methodology, analysis, and reporting of NGS results, establishing best practices for data sharing in line with General Data Protection Regulation and for advising on assessment of clinical utility of NGS-based testing [54]. RWD-based studies respecting these guidelines should address payer and regulatory-body concerns regarding RWE, and thus aid their incorporation into value assessment frameworks. Current frameworks are lacking in useful examples of RWE to demonstrate value, and there is a clear need for expert panel discussions to provide these. Guidelines on how best to report RWD-based studies in medical journals are also needed, with few journals currently providing such recommendations [113].

## 5. Conclusions

Therapy based on individual tumor molecular biomarkers is a promising way to improve cancer treatment and a powerful demonstration of the value of precision medicine, with potential across a broad spectrum of care.

General and country-specific recommendations can be taken from our series of expert panel discussions to address the challenges facing implementation of NGS across Europe, in both the short and the long term. Overall, multi-stakeholder engagement is needed to equalize patient access to health systems, providing analytically and clinically validated advanced diagnostics.

The principal specific recommendations, many of which should be supported by the EU review of pharmaceutical legislation, Europe’s BCP, IVDR, EHDEN, and Cancer Mission, are:

1. Evidence generation demonstrating the clinical value of NGS through traditional and innovative clinical trials and standardized, guideline-driven RWD-based studies.
2. Converging approval of diagnostics and medicine to secure availability.
3. A dynamic, collaborative, and multi-stakeholder national precision-medicine strategy that is aligned across Europe, with European collaboration.
4. Up-to-date guidance specifying when and where NGS should be performed, along with minimum standards for testing. Updating the European Commission’s 2003 recommendations on cancer screening [114] to reflect the challenges detailed in this review is critical for providing proper guidance and establishing equitable early diagnosis through NGS.
5. Integration and ongoing update of national guidelines covering NGS testing, in collaboration with scientific medical societies.
6. Use of MTBs and clinical decision-supporting systems to align treatment strategies, based on profound expertise in cancer genomics.
7. Increased awareness and education of stakeholders, including physicians, patients, and payers.
8. Standardized value assessment frameworks that incorporate values from the perspective of multiple stakeholders.
9. Sufficient and consistently adjusted budgeting for molecular testing, with significant investment in bioinformatics and artificial intelligence-based infrastructure for NGS data storage, analysis, and interpretation.
10. Depending on the country-specific conditions, centralized, standardized centers with laboratories performing high-quality, in-house, and commercial NGS, or professional laboratories offering end-to-end services.
11. Standardized, pan-European, and centralized multi-stakeholder registries collecting RWD related to diagnosis, treatments, and outcomes, with consensus guidelines at the European level to standardize RWD-based studies.
12. Utilization of horizon-scanning techniques to identify future challenges related to innovative testing techniques, such as NGS, thus preparing stakeholders for their implementation into healthcare [115].

## Figures and Tables

**Table 1:** Country-specific MTB usage and recommendations across Europe.

Country	MTB Usage	Recommendations
Belgium	Most large centers have MTBs, and precision projects to initiate national MTBs have been started, which will enable the exchange of expertise. In general, MTBs tend to be convened on an ad hoc basis and attendance is irregular, e.g., oncologists may not always attend	Key performance indicators for MTB meetings to be defined
Bulgaria	The decision to conduct biomarker screening is made by interdisciplinary MTBs	
France	MTBs are required and mandatory for decision-making concerning all oncology patients (public and private hospitals) and take place in a health facility, a group of health establishments, an oncology network, or as part of coordination centers in oncology (3C). The organizational procedures for MTBs are defined by article D. 6124-131 of the Public Health Code. MTBs must be carried out in the presence of at least three physicians from different specialties working with patients, providing a relevant opinion on all the procedures envisaged. The doctor then informs the patient and gives a personalized program of care. For complicated cases, local, regional, or national molecular MTBs are available and can connect to early-phase clinical trials. Fifteen “national reference networks for rare adult cancers” have been labeled by the French National Cancer Institute and provide national MTBs	Facilitate access to the national reference networks. Analyze obstacles in cross-border cancer treatment. Inform citizens of their rights with regards to cross-border treatment
Germany	Decision for large-scale NGS is dependent upon MTBs, which are integrated at most, if not all, university hospitals. Hospitals in Germany run weekly MTBs on a national and international scale (600–1000 patients per year, per site). MTBs typically have a structure for reporting the patient-decision process and requirements for data storage. The German government has encouraged health insurance companies to provide significant funding for translational projects at universities, including the roll out of MTBs, which means there is a network of working groups as well as a standardization of reporting and quality control. At least 100,000 patients in Germany qualify for CGP every year and the current system is not scalable, as due to sectoral limitations in patient care, most tumor samples and cases are not transferred into centralized genome centers for in-depth molecular analysis and MTB-based therapy evaluation	To support the large number of patients, MTBs may need to be reinvented to be connected with early clinical trials and automated with bioinformatics and artificial intelligence-based support. Guidance to develop fit-for-purpose MTBs is also critical
Israel	MTBs are not overburdened and are conducted in an efficient manner, with a broad spectrum of expertise. MTBs are weekly/bi-weekly 2 h sessions that cover every tumor type; there has been an MTB in Israel (Hadassah) running for more than 8 years, and it has become partially virtual to allow sharing of expertise between local hospitals. There are no strict rules on the required composition of an MTB, although they must include pathologists, oncologists, geneticists, and radiotherapists. Referral to MTBs is increased when discussions are virtual; recommendations are provided to physicians to refer the patient to a local MTB (the process is not regulated). Some cases may then be sent to Foundation Medicine, Inc., for further discussion	Share best practice to establish a cross-country-accepted framework in terms of the required composition of MTBs
Italy	Italy is currently working to create a national MTB in collaboration with regional MTBs. A national agency, in charge of evaluating the efficiency of regional healthcare systems, is attempting to provide national guidelines for regional MTBs. Some regions (such as Regione Veneto) have also provided individual guidance for MTBs (e.g., structure, criteria to access molecular profiling, access to off-label therapies, reimbursement procedures). However, not all hospitals are able to incorporate the required expertise, and these hospitals should defer to centralized MTBs to discuss complex patient cases. MTBs in Italy use the ESCAT and OncoKB scales (for less frequently reported genetic variants) to guide treatment decisions; only targeted therapies with a specific level of evidence (Level 1 or 1A) can be recommended. MTBs have a strict procedure for obtaining access in order to limit patient numbers, with access only recommended by multidisciplinary boards for particular tumor types (and not by physicians or patients). Some precision medicine trials are ongoing in the country	MTBs should combine both high-quality clinical and genetic expertise to place the NGS results into a clinical context. Establish EU-wide guidelines for MTBs
The Netherlands	Determining eligibility for MTBs is performed on a case-by-case basis	
Poland	Typically, three teams are involved in MTBs, with representatives from radiology, pathology, and clinical teams (the clinical team is often the most important team, as they provide an overview of all patient information). Clinically, there are low numbers of molecular diagnostic and genetic experts in Poland, challenging the implementation of MTBs	
Portugal	The Portuguese Oncology Institute of Porto was the first to implement an MTB in Portugal; the MTB typically includes medical oncologists, pathologists, and molecular geneticists/pathologists, in addition to the treating physician	
Republic of Ireland	Although use of MTBs is variable (Republic of Ireland does not currently have a high number of patients included in MTBs), they enable international collaboration and are useful in rare cancer types. Some clinical colleagues use international (e.g., co-operative group) MTBs, most commonly as part of research or in clinical trials, and there are initiatives to facilitate organization of MTBs	Additional resources and integration, along with ongoing education, required for mainstreaming of MTBs
Slovenia	MTBs are established in large cancer centers. Molecular testing is performed based on ESCAT guidelines. All additional indications for NGS testing are made by MTBs	
Spain	No national MTBs, only regional, and these mainly occur within large hospitals that use NGS	
Sweden	Diagnostics and the treatment of cancer are driven by firm clinical guidelines, which involve MTBs and the treating clinicians	
United Kingdom	MTBs are uncommon but will evolve as further NGS data become available, especially with the roll-out of genomic hubs by NHS England	

CGP, comprehensive genomic profiling; ESCAT, ESMO Scale for Clinical Actionability of molecular Targets; ESMO, European Society for Medical Oncology; EU, European Union; MTB, molecular tumor board; NGS, next-generation sequencing; NHS, National Health Service.

## Data Availability

Not applicable.

## Supplementary Materials

The following supporting information can be downloaded at: www.mdpi.com/article/10.3390/jpm12010072/s1, Table S1: Country-specific NGS governance challenges and recommendations across Europe; Table S2: Country-specific NGS clinical standardization challenges and recommendations across Europe; Table S3: Country-specific NGS stakeholder awareness and education challenges and recommendations across Europe; Table S4: Country-specific NGS reimbursement and subsequent access challenges and recommendations across Europe; Table S5: Country-specific NGS centralization and infrastructure capacity across Europe; Table S6: Country-specific RWE challenges and recommendations across Europe.
