# Supplementary material for: Identifying the Steps Required to Effectively Implement Next-Generation Sequencing in Oncology at a National Level in Europe
Source: J Pers Med. 2022 Jan 8;12(1):72. doi: 10.3390/jpm12010072 (PMC8780351; doi:10.3390/jpm12010072)
Supplement: Supplementary file 1 [file jpm-1523001-supplementary.pdf › jpm-1523001-supplementary.pdf]

SUPPLEMENTARY APPENDIX

Table S1. Country-specific NGS governance challenges and recommendations across Europe.

**Country**	**Challenges**	**Recommendations**
Bulgaria	Only specific centers of excellence perform NGS, although there is a still a lack of harmonization between them (e.g., in terms of infrastructure and procedure)	• European guidelines are required, particularly for private laboratories
France	Lack of harmonization in NGS (i.e., test type, panel size) between public and private laboratories, with no national guidelines. Many private laboratories each present with their own challenges (e.g., budget, goal), limiting implementation of NGS and leading to use of sequential molecular tests that have a quicker turnaround time. Funding of molecular tests through the repository of innovative acts outside the nomenclature (RIHN) partially covers the cost of the tests, which remain the responsibility of health establishments. This can lead to unequal access to care	• National recommendations are required.<br>• The RIHN system must be reformed through a reimbursement by social security as part of routine care.<br>• Share best practice to establish a common EU framework for NGS
Germany	Although there are national personalized medicine centers, there are challenges with public access to datasets from these centers and with data management (e.g., collection, storage, harmonization). Generating platforms for integration of datasets from different centers is also a key challenge. Challenges may be aided by an incoming law stating that all patients with cancer should receive whole-genome sequencing. Directly from the Ministry of Health, the new law has bypassed all stakeholders,	• Collaboration with stakeholders is key to address their specific needs

	and has huge implications on overall healthcare, testing infrastructure, insurance companies, and caregivers. A Ministry-funded program, "genomDE," is now starting and will provide the infrastructure for clinical genome sequencing in rare diseases, personalized medicine, and cancer. Implementation into the clinic is anticipated to start in 2023.	
Israel	There are very few healthcare providers, leading to challenges in initiating a national database, compounded by local restrictions regarding data privacy and sharing	
Italy	Lack of harmonization in NGS (i.e., test type, panel size) between public and private laboratories. Although there is a validated, national platform to improve patient access to large NGS panels, this is challenged by the requirement for test accreditation, as many tests will not be of the required utility and validity. This has increased reliance of laboratories on commercial-test providers. Furthermore, there have been ~15 intersociety recommendations for the implementation of NGS in the last 2 years; although there is agreement between the different societies, implementation of such recommendations and harmonization of the process across regions by the government are difficult, particularly due to regional differences in healthcare and reimbursement	• European frameworks are critical to address the lack of harmonization
The Netherlands	There are committees that determine diagnostics and treatment plans for different diseases, including cancer. These committees are well organized and are aligned across hospitals. However,	

	this limits flexibility and means the process must be revised if there are new developments in the field. There are no significant regional differences in NGS testing across the Netherlands, due to the small size of the country, with large academic centers covering each region	
Poland	Specialist genetic and molecular laboratories do not exist across all hospitals. Furthermore, although recommendations for NGS implementation from the Ministry of Health, government representatives, and policymakers will be published in Poland soon, implementation is challenging, particularly for small local hospitals (larger cancer centers have good laboratories and financing systems in place). Certification, which is required for NGS testing in Poland, is also more difficult for smaller local hospitals, due to the financing, equipment, and expertise needed	• Education of medical professionals is key; current laboratories require additional clinical information, including molecular diagnosis, to identify predictive information, treatment, and prognostic factors.<br>• To resolve financing challenges with the National Health Fund, it is critical to demonstrate that reimbursement of advanced diagnostics is cheaper for the healthcare system, as only patients with good overall clinical outcomes are selected
Republic of Ireland	NGS is conducted regionally and is fragmented at the national level, and there is a requirement for harmonization of research infrastructure. There is some degree of regulatory framework, but it is not mainstream. A national cancer genomics strategy has begun to aid harmonization	• Coordination of a public genome project like the United Kingdom's 100,000 Genomes Project
Slovenia	While there is a national cancer control plan, there is a struggle to include precision medicine, which would make financing easier	• Work with national health authorities to make them aware of the importance of including precision medicine in their national cancer plans
Southern European countries (e.g., Greece)	Despite the presence of national precision-medicine networks, there is a lack of understanding of the initiatives among governments, limiting input from experts	• Education of government regarding national precision medicine initiatives.<br>• Involve policymakers in the debate

Spain	Only regional personalized medicine initiatives exist and there is heterogeneity between these initiatives	• Advocate for EU initiatives on personalized medicine
United Kingdom	There are different tracks of governance between Scotland and England, leading to differences in the approval of MGTOs. There is also a reluctance of payers to engage in conversations regarding NGS, largely due to budgetary implications, and the separation of research from clinical service, with limited engagement and reimbursement for research. This has been aided by the April 2021 roll out of genomic hubs by NHS England, although is still a work in progress for the devolved nations	

EU, European Union; MGTO, molecularly guided treatment option; NGS, next-generation sequencing; NHS, National Health Service.

Table S2. Country-specific NGS clinical standardization challenges and recommendations across Europe.

Country	Challenges	Recommendations
Belgium	The process of standardization is underway, but there is still variation between laboratories, and this will become a key challenge for data exchange in the future	
France	NGS methodologies/techniques and interpretation of results are not harmonized between institutes	• EQA initiatives and networking guidelines for the "must" panels and the "clinical-research oriented" panels
Germany	There are working groups to discuss NGS standardization and laboratories are accredited by standardized national bodies. Laboratories also use the Logical Observation Identifier Names and Codes (LOINC) database to ensure harmonized result reporting and interpretation [1]. Key challenges in Germany are the absence of guidance on evidence requirements to demonstrate the clinical utility of large gene panels and the lack of personalized treatment data from routine clinical practice (i.e., dose, duration, efficacy, safety, outcomes); for most of these patients, there are no data from clinical trials. Generating such data is expensive and is therefore not conducted widely. Transcriptomics, which could be useful to interpret genome data, is not yet reimbursed by health insurance companies	• Evidence demonstrating the clinical utility of NGS in different tumor types<br>• Clear guidance stating the patient populations and NGS panels that should be included is key
Greece	There are research institutes that perform NGS, but methodologies/techniques and interpretation of results are not harmonized	
Italy	Different models of accreditation are used by different laboratories. Due to a lack of organization, there is also a lack of	

	compliance in Italy towards standardization and harmonization of the NGS workflow	
The Netherlands	Diagnostic laboratories in the Netherlands require ISO 15189 accreditation for NGS, leading to good standardization and validation of test/workflow. Variations may still be introduced in the pre-analytical phase, and standards may not be harmonized; however, this may be aided by the incoming new version of the ISO 15189 accreditation	• Patients should request their test to be conducted in an accredited laboratory, to ensure proper diagnosis and management.<br>• There are standards in the Netherlands for the pre-analytical phase that are required to be followed and tracked; this should be extended for the entire NGS workflow
Poland	There is a national certification for NGS methodology (not for laboratories), but this certification is very expensive; therefore, access is limited for smaller public laboratories	• Centralized test providers may prove useful in this context
Portugal	Most hospitals are attempting to implement NGS, but each hospital uses different NGS panels, limiting standardization, and comparisons	
Republic of Ireland	A key challenge is oversight of clinical standardization, particularly when samples are sent to commercial providers for larger comprehensive testing	• A national governance structure, with minimum standards for testing, is required
Spain	The SEAP-IAP quality assurance program in pathology aims to ensure quality and standardization of molecular testing (in some cases, NGS) [2]. However, as participation is voluntary, adherence is poor. Other than SEAP-IAP, there are no coordinated initiatives that enable similar test methodologies and result interpretation, leading to variation in trust for different laboratories	• Laboratories must be accredited for NGS.<br>• Work with national health authorities to set up accredited laboratories for NGS

EQA, external quality assessment; ISO, International Organization for Standardization; NGS, next-generation sequencing; SEAP-IAP, Spanish Society of Pathological Anatomy – International Academy of Pathology.

Table S3. Country-specific NGS stakeholder awareness and education challenges and recommendations across Europe.

**Country**	**Challenges**	**Recommendations**
Germany	Physicians are not convinced that NGS obtains sufficient actionable results for individual patients (excluding patients with lung cancer) and adds further benefit to other multi-panel gene testing techniques. Payers have a good understanding with healthcare providers and are eager to reimburse patients that have been evaluated by MTBs. Indeed, less than 5% of all patients seen in MTBs, as part of centers for personalized medicine, do not receive NGS reimbursed from an insurance company. Patients receiving NGS reimbursement are typically also reimbursed for treatment, if appropriate	• All information is needed, even if the information is not useful for the individual patient, and patients must be informed of the comprehensive view of genomic testing, as well as its future, rather than immediate, benefit. Patient advocates are key for this education.<br>• Education of medical oncology students is very important to adhere to patient needs; all oncologists must understand the meaning of precision oncology and the low chances of benefit for the individual patient
Israel	Peripheral patient awareness of the value of NGS is more limited than in the large tertiary centers. To improve patient engagement in NGS, oncologists are currently describing NGS as a technique to reduce the need for chemotherapy	• Establish collaboration among national and EU patient organizations to raise awareness on the value of NGS
Italy	There is an over-expectation among patients regarding the value of NGS	• For patients that may benefit from NGS (e.g., those with lung cancer), the technology must be optimized.<br>• Clinicians, patient advocacy groups, and the media must be educated on the value and utility of NGS.<br>• It is key to accept a higher degree of uncertainty in the field of precision oncology, as use of gold-standard randomized clinical trials is limited
Poland	Oncologists have a good knowledge of molecular diagnostics as treatment decisions, and consequently NGS, are generally based	• Education of clinicians is very important, particularly regarding the use of NGS for prognosis.

	on this (but not for all cancers). Payers and patients are also aware that molecular diagnostics (not necessarily NGS) are required to achieve benefits of innovative therapies, and Poland has prepared special recommendations for payers, the Ministry of Health, and the government	• Financial management and education of the hospital performing NGS is important and requires cooperation between large cancer centers. In Poland, such education began in 2016 (when preparation for a molecular diagnostics project began)
Republic of Ireland	There is a good awareness of NGS in the oncology community, although skepticism regarding the value of NGS beyond lung cancer remains	• Evidence demonstrating clinical utility of NGS beyond lung cancer, using both clinical trials and RWE, is key
Slovenia	There is insufficient knowledge of the applicability and usefulness of NGS in routine clinical practice amongst multiple stakeholders	• Education of stakeholders is key to raise awareness of NGS
Sweden	Although awareness of NGS among stakeholders is good, implementation of NGS into clinical oncology is still slow due to a lack of supporting clinical utility data	• Evidence demonstrating clinical utility of NGS, using both clinical trials and RWE, is key

EU, European Union; MTB, molecular tumor board; NGS, next-generation sequencing; RWE, real-world evidence.

Table S4. Country-specific NGS reimbursement and subsequent access challenges and recommendations across Europe.

Country	Challenges	Recommendations
Belgium	There is a specific NGS budget each year, and laboratories must pay the remaining deficit. Insufficient budgets dedicated to NGS are also a challenge. Private insurance does not routinely cover NGS	• As mentioned by the ESMO recommendations, discussions between the physician and patient, including expectations of NGS and possible consequences, are critical
Bulgaria	Only companion diagnostics are fully reimbursed, and other tests are dependent on the pharmaceutical company or hospital (and if not, the patient) covering the cost. There is no program for reimbursement of NGS, so it is reliant on collaborations between private companies and pharmaceutical companies (otherwise, the patient must pay). Bulgaria also has a limited capacity and experience/expertise for HTA processes for NGS, which slows down reimbursement	• Advocate for harmonized and transparent reimbursement rules across the EU
France	Reimbursement is the main bottleneck to implementation of NGS in France. There is a reimbursement system, funded by the French Ministry of Health, and reimbursement is related to the size of the testing panel(s). However, the budget for NGS is constant, which means that as the gene panel increases with the number of genes to be tested, reimbursement drops, and additional funding is required. For example, even though NGS will be mandatory in most early	• NGS should be systematically reimbursed across tumors. Inspiration should be taken from Spain, in which there is better collaboration with pharmaceutical companies, thus improving reimbursement and testing possibilities

	stage cancers to identify whether immunotherapy or targeted therapy is more appropriate (e.g., in stage I–IIIA lung cancer by the end of 2021), NGS will still not be fully reimbursed (only at ~50%). The situation varies between public and private laboratories	
Germany	NGS is reimbursed in ~90% of patients; however, infrastructure (e.g., paperwork, case management, documentation, genomic counseling, central data storage) for testing is not reimbursed currently. Reimbursement status varies between hospitals, although this should be aided by the incoming new law in Germany stating that all patients should receive whole-exome/-genome sequencing. To support the evidence generation, Germany has included outcome data in a centralized data pool, accessible by all university hospitals and facilitated by the German Research Fund. This is key to raise patient acceptance of NGS and therefore increase pressure on clinicians to use the technology. However, even with such evidence generation, hospitals may be reluctant to transfer budget to NGS, due to a lack of trust	• Further guidelines are required to mandate use of the data pool outside university hospitals, and the European Cancer Patient Coalition and national representatives are key in raising awareness on the benefits of using NGS.<br>• Laboratory education of physicians in interpreting NGS results is key to ensure that the data are actionable and increase public trust in molecular testing, which has been reduced as a result of COVID-19 testing.<br>• There is a need to work on future reimbursement of whole transcriptome analysis in cancer treatment
Israel	CGP is available and publicly funded for adults with non-small cell lung cancer, colorectal cancer, carcinoma-of-unknown-primary-origin, and urinary tract cancer, and all pediatric patients with solid and non-solid advanced malignancies. Budget is allocated	

	for every test, and commercial large-panel tests are used	
Italy	Reimbursement is region-dependent; NGS is not considered “essential care” so is not fully reimbursed by the national healthcare system. Also, many patients with cancer require multiple NGS tests and re-biopsies, which are not covered. Liquid biopsies, although not reimbursed themselves, are paid for by the national healthcare system using a group of codes including extraction, sequencing, and analysis of genetic material, which is key when NGS must be performed on both tissue and blood (e.g., in lung cancer). By the end of 2021, it will be mandatory for laboratories in Italy to be CLIA-accredited. Many laboratories are currently not CLIA-accredited, limiting reimbursement of therapies and increasing laboratory reliance on commercial NGS testing. Many hospitals, particularly in the public sector, require reimbursement codes for testing, limiting their implementation	• The patient populations that require NGS must be defined to manage cost implications, and it is key to involve patient advocacy groups in this process.<br>• Value assessment frameworks must include both cost-effectiveness analyses and patient preferences, particularly considering that cost-effectiveness may vary between tumor types.<br>• Somatic and germline tests must be distinguished when considering reimbursement, as they present different processes and challenges
The Netherlands	Reimbursement in the Netherlands is not a huge challenge; if the multidisciplinary team decide that NGS is required for the patient, it will be reimbursed. Testing is normally included in the care plan and reimbursed as part of the treatment. However, some institutes are attempting to cut costs, which may limit	

	reimbursement. Replacing all equipment as part of compliance with IVDR will also present a financial challenge. Based on results from the WIDE study demonstrating clinical-grade whole genome sequencing to be feasible in routine molecular diagnostics in a comprehensive cancer setting [3], law-makers in the Netherlands have backed a proposal for whole genome sequencing to be funded by the public healthcare system	
Poland	Advanced diagnostic tests are more expensive than simpler tests. The Ministry of Health in Poland has published recommendations for diagnostics for lung cancer only; diagnostics of other tumor types rely on recommendations from the Scientific Society	• From a patient perspective, as part of diagnosis and treatment, it is important to use NGS to create a "genomic passport." The genomic information required varies between tumor types, and clinical and technological recommendations are needed.<br>• Without recommendations for quality, it is challenging to develop a good strategy for NGS reimbursement and implementation.<br>• NGS should be harnessed as a companion diagnostic to obtain greater reimbursement
Republic of Ireland	Medium-sized gene panels with standard actionable alterations are reimbursed by the public healthcare system, whereas more comprehensive panels are widespread in the private system (i.e., out-of-pocket expenses). A subgroup of the national cancer control	• A consistent, accessible, and transparent framework is required to be generated whereby access to, when relevant, and reimbursement of more comprehensive molecular profiling is needed

	program (Cancer Molecular Diagnostics [Drugs] Advisory Group) are developing recommendations for larger panels, but these are yet to be utilized	
Spain and Portugal	No powerful tools for the reimbursement of advanced diagnostics (everything is driven by therapy), although some diagnostics in Spain have been reimbursed purely based on prognostic data (e.g., Oncotype DX). There is reimbursement for specific mutations in Spain (e.g., *EGFR* and *ALK* in lung cancer); however, there is heterogeneity across regions in tests conducted and available NGS technology. Although there is excitement regarding genetic testing in Spain, reimbursement is low, limiting translation into clinical use	
United Kingdom (Scotland)	There is no formal reimbursement for testing, and laboratories are given an annual budget to deliver the service required. This does not enable flexibility and limits the development of new technology, increasing reliance on commercial companies	

CGP, comprehensive genomic profiling; CLIA, Clinical Laboratory Improvement Amendments; ESMO, European Society for Medical Oncology; EU, European Union; HTA, health technology assessment; IVDR, In Vitro Diagnostic Regulation; NGS, next-generation sequencing.

Table S5. Country-specific NGS centralization and infrastructure capacity across Europe.

Country	Challenges	Recommendations
Belgium	Agreement between the central health agency and molecular pathology laboratories settles reimbursement of molecular testing, provided the results are stored in a central database	• Provide public access to database for research and RWE purposes
Bulgaria	In-house platforms are not well implemented, due to inadequate bioinformatic specialists	• Further specialized training of geneticists and bioinformaticians is required
Germany	IT infrastructure and harmonization of this across Europe is a key challenge but is critical for data collection and analysis. Data accessibility is another challenge; local data cannot be accessed by other regions and current datasets are siloed, leading to further challenges. Test report interpretation with limiting existing capacity and insufficient resourcing for a specialized workforce and implementation of adequate training are also important challenges. In Germany, testing infrastructure is not centralized (rather, federated) and although there are attempts to coordinate testing at the regional level, this is only in specific tumor types, and the capacity and infrastructure to perform NGS testing is not nationwide (in particular, not in the countryside)	• IT infrastructure requires an initial significant bioinformatics investment (although it would be cost-efficient in the long term).<br>• Bioinformaticians are key to aid data analysis and management, although this is not possible in every hospital/oncology clinic, stressing the need for MTBs in comprehensive cancer centers. MTBs in smaller hospitals are not preferred, as they may not contain all the expertise required.<br>• Although centralization is key, academic centers are still important for broad knowledge generation, and public–private partnerships are important for routine healthcare services and ensuring connectivity between local hospitals.<br>• To aid use of artificial intelligence, it is important to standardize and prepare the datasets that artificial intelligence algorithms could be

		applied to. Although artificial intelligence has been utilized in Germany to aid data sharing and access, current datasets are not harmonized
Israel	Availability of bioinformatics technology, test report interpretation with limited existing capacity and insufficient resourcing for a specialized workforce and implementation of adequate training, remain challenges in Israel	• Given the large amount of data from NGS, assistance (e.g., from MTBs, clinical decision-support systems) is required to aid interpretation of results and therefore treatment decisions
Italy	There is a decentralized system, which may mean institutions use their own NGS tests and may not generate reliable results. There are also no recognized bioinformaticists that can work in NGS labs, requiring additional payments for informatics. Political challenges also exist in Italy, as infrastructure can only be developed at regional and national government levels	• Depending on expertise available, local hospitals should refer to centralized MTBs, arranged and facilitated by the government.<br>• Harmonized NGS platforms and infrastructure would be useful for future data sharing
The Netherlands	There is a model of a public centralized system, which appears to be successful for peripheral hospitals, although raw data are stored for a short period of time only and insufficient funding remains a challenge	
Portugal	Main challenges include insufficient funding for NGS and insufficient resourcing of a specialized workforce or implementation of adequate training	• Using high-quality, commercial NGS platforms already available would enable increased standardization; however, they are more costly, limiting usage across all countries (including

		Portugal). Thus, in-house platforms demonstrate greater accessibility and use
Republic of Ireland	The system is semi-centralized, and NGS tests are mainly carried out for lung, colon, gastrointestinal stromal tumors, and melanomas; broader use is an issue. There is sufficient capacity for basic NGS testing, but not for more advanced tests and clinical diagnostics, and there is controversy regarding genomic data storage	• Additional infrastructure is required, along with bioinformatics support, which could be located within more centralized facilities (particularly for advanced molecular analyses)
Slovenia	Availability of IT and a specialized workforce, especially bioinformaticists and geneticists, is limited. Therefore, commercial NGS platforms are mainly used	
Sweden	There is a need to invest in infrastructure; much of the NGS analysis is done outside the country. Genomic Medicine Sweden is a national genomic initiative aiming for implementation of large-scale sequencing techniques and genomics in healthcare for patients with rare diseases and cancer [4]	
United Kingdom	The roll out of genomic hubs by NHS England enables regional access to NGS data for some tumor types [5]. However, in Scotland, infrastructure is already centralized, and capacity is mostly present, although there are currently no discussions regarding data management and storage	

IT, information technology; MTB, molecular tumor board; NGS, next-generation sequencing; NHS, National Health Service; RWE, real-world evidence.

Table S6. Country-specific RWE challenges and recommendations across Europe.

Country	Challenges	Recommendations
France	RWE quality depends on sample and laboratory quality, and there is a discrepancy between RWE at the large organizational level and in local hospitals. Promises made to patients regarding RWE may not always be fulfilled. Currently, RWE in the context of large panels (>500 genes) is useful for research and networking, but not for clinical practice. These large panels also require high quantities of sample material, which may lead to false-negative results	
Germany	Data standardization and harmonization are key challenges to address but are critical to generate sufficient RWE for enough patients and enable clinically meaningful conclusions. There is a network in Germany working to develop national recommendations for RWE collection and address RWE standardization. Data ownership is also a challenge, leading to limited data sharing, as well as biased RWE, resulting in limited high-quality data	• Networks in different countries should collaborate to align RWE recommendations across Europe.<br>• Datasets, with integrated genetic and clinical data, should be openly accessible and shared regionally, nationally, and internationally.<br>• Further information from the European Alliance for Personalised Medicine is needed on disease prevention
Israel	RWE is used to monitor the effect of the healthcare system (i.e., the hospitals) and to supplement clinical trials	• The aim and end goal of RWE must be defined

Portugal	There are attempts to develop national registries to improve evidence generation, with input from hospitals and funding from the government, but the process is only in its infancy. Attempts are also being made to collect and establish RWE (patient quality of life) as part of the evaluation of the patient perspective in value assessment frameworks	• Patient organizations should advocate for the EU to support Member States to develop comprehensive national registries
Slovenia	There is a long-lasting tradition of national cancer registries in Slovenia (e.g., the Cancer Registry of Republic of Slovenia), and there are attempts to facilitate more profound RWE data collection (including molecular data) for melanoma and lung cancer via these registries. Data standardization and uniform collection are the main challenges	
Spain	There are issues with registries being able to admit patients from clinical trials	

EU, European Union; RWE, real-world evidence.

References

1. McDonald, C.J.; Huff, S.M.; Suico, J.G.; Hill, G.; Leavelle, D.; Aller, R.; Forrey, A.; Mercer, K.; DeMoor, G.; Hook, J., et al. LOINC, a universal standard for identifying laboratory observations: a 5-year update. *Clin Chem* **2003**, *49*, 624-633.
2. Spanish Society of Pathological Anatomy – International Academy of Pathology. Who we are. Available online: https://www.seap.es/quienes-somos (accessed on 29 September 2021).
3. Monkhorst, K.; Samsom, K.; Schipper, L.; Roepman, P.; Bosch, L.; de Bruijn, E.; Hoes, L.R.; Riethorst, I.; Schoenmaker, L.; van der Kolk, L., et al. Validation of whole genome sequencing in routine clinical practice. *Ann Oncol* **2020**, *31*, S784. Abstract 1189O.
4. Edsjö, A.; Friedman, M.; Rosenquist, R. Genomic Medicine Sweden - a national initiative for the broad introduction of precision medicine in Swedish healthcare [article in Swedish]. *Lakartidningen* **2021**, *118*, 21023.
5. NHS England. Genomic Laboratory Hubs. Available online: https://www.england.nhs.uk/genomics/genomic-laboratory-hubs/ (accessed on 29 September 2021).
